# Cinchonine and cinchonidine alleviate cisplatin‐induced ototoxicity by regulating PI3K‐AKT signaling

**DOI:** 10.1111/cns.14403

**Published:** 2023-08-14

**Authors:** Dongmei Tang, Xue Wang, Jingfang Wu, Yimeng Li, Cai Li, Xiangyun Qiao, Li Fan, Yutao Chen, Huanhuan Zhu, Zhiyuan Zhang, Yingzi He

**Affiliations:** ^1^ ENT Institute and Department of Otorhinolaryngology, Eye & ENT Hospital, State Key Laboratory of Medical Neurobiology and MOE Frontiers Center for Brain Science, NHC Key Laboratory of Hearing Medicine Fudan University Shanghai China; ^2^ Department of Otorhinolaryngology‐Head and Neck Surgery First Affiliated Hospital of Nanchang University Nanchang China; ^3^ Department of Otorhinolaryngology Head and Neck Surgery The Second Affiliated Hospital of Anhui Medical University Hefei China

**Keywords:** apoptosis, cinchonidine, cinchonine, cisplatin, ototoxicity

## Abstract

**Aim:**

Cinchonine (CN) and its isomer cinchonidine (CD), two of the common cinchona alkaloids, are wildly used as antimalarial drugs. However, the effects of CN and CD on the auditory system are unknown.

**Methods:**

Molecular docking and molecular dynamics (MD) simulation were used for predicting effective drugs. The CCK‐8 assay was conducted for assessing cell viability in House Ear Institute‐Organ of Corti 1 (HEI‐OC1) cells. MitoSox Red staining revealed reactive oxygen species (ROS) amounts. TMRM staining was used to assess the mitochondrial membrane potential (ΔΨm). Immunofluorescence staining of myosin 7a was used to examine hair cells (HCs) in cisplatin‐treated neonatal mouse cochlear explants, while TUJ‐1 immunostaining was used for the detection of spiral ganglion neurons (SGNs). Cleaved caspase‐3 and TUNEL immunostaining were utilized for apoptosis assessment. Immunoblot was carried out to detect PI3K‐AKT signaling effectors.

**Results:**

Pretreatment with CN or CD significantly increased cell viability and reduced mitochondrial dysfunction and ROS accumulation in cisplatin‐treated HEI‐OC1 cells. Immunofluorescent staining of cochlear explants showed that CN and CD attenuated cisplatin‐induced damage to SGNs and HCs. Immunoblot revealed that CN and CD downregulated the expression of cleaved caspase‐3 and activated PI3K‐AKT signaling in cisplatin‐injured HEI‐OC1 cells.

**Conclusion:**

CD and CN can reduce ototoxicity caused by cisplatin and might help treat cisplatin‐associated hearing loss.

## Graphical abstract

Cisplatin induces the accumulation of ROS in HCs, which inhibits the PI3K/AKT signaling pathway and leads to apoptosis of hair cells. Pretreatment with cinchonine (CN) and its isomer cinchonidine (CD) activates the PI3K/AKT signaling pathway through stable connection to PIK3CA, thereby inhibiting the accumulation of ROS induced by cisplatin exposure and suppressing the apoptosis of HCs. In conclusion, CN and CD protect hair cells against the ototoxic damage of cisplatin by regulating the PI3K‐AKT pathway.

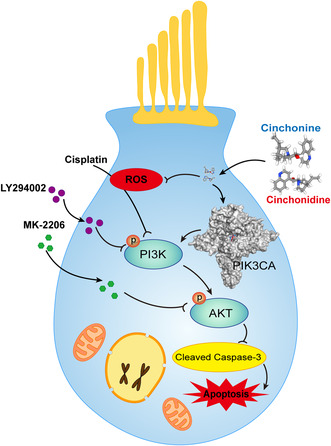

## 1 INTRODUCTION

Cisplatin represents a chemotherapy drug commonly utilized in clinical practice for treatment of diverse cancers such as carcinomas, germ cell tumors, sarcomas, and more.^1^ However, cisplatin induces ototoxicity in ~62% of patients, resulting in irreversible sensorineural hearing loss.^2^, ^3^ Previous studies have reported that cisplatin directly damages the organ of Corti and spiral ganglion both in vivo and in vitro.^4^, ^5^ Mechanisms contributing to cisplatin‐associated hearing loss include oxidative stress, DNA damage, inflammation, and activation of cell death pathways, which involve apoptosis, necroptosis, and ferroptosis.^6^, ^7^, ^8^, ^9^ No potent drugs or therapeutic approaches are presently available for cisplatin‐related hearing loss.^10^ Therefore, there is a pressing need to develop agents with protective effects on HCs and SGNs as a treatment option for cisplatin‐induced hearing loss.

Cinchonine (CN) and its isomer cinchonidine (CD) are generated from the bark of a tree called Cinchona.^11^ CD and CN are utilized for malaria treatment and as anti‐multidrug resistance medications in different tumors.^12^ CN also possesses antitumor,^13^ antiplatelet,^14^, ^15^ and anti‐obesity^16^ properties. The anti‐obesity properties of cinchonine are mediated by suppressed inflammation in fat tissue.^16^ Cinchonine was shown to inhibit neutrophil chemotaxis and oxidative stress response.^17^

Phosphoinositide‐3‐kinase (PI3K) signaling is crucial in cochlear hair cell and neural development,^18^, ^19^ and protein kinase B (AKT) also regulates auditory HC survival and protects auditory HC from gentamicin‐induced toxicity.^20^, ^21^ Recent evidence indicates that PI3K‐AKT signaling could be used as a therapeutic target for harmful stimuli such as the ototoxic drugs gentamicin and cisplatin as well as cell survival induction.^22^, ^23^ In noise‐induced cochlea damage, deferoxamine triggers mesenchymal stem cell homing via PI3K‐AKT pathway induction.^24^ We selected 17 small molecule drugs from FDA‐approved Chinese drugs and bound them to the PIK3CA (PDB ID:4a55) protein by molecular docking and used the protein–ligand binding energy as a reference to screen effective drugs for subsequent validation. We found that CN and CD could interact with PIK3CA to produce stable binding complexes; their stability was also verified via MD simulations. We hypothesized that CN and CD reduced cisplatin‐induced ototoxicity via PI3K‐AKT signaling.

To test our hypothesis, HEI‐OC1 cells and mouse cochlear explants were examined as in vitro models. We found that CN and CD could reduce hair cell apoptosis through regulating PI3K‐AKT signaling, specifically by reducing mitochondrial ROS production and protecting mitochondrial function. This study provides a novel alternative therapeutic option for preventing cisplatin‐associated ototoxicity.

## 2 MATERIALS AND METHODS

### 2.1 Molecular docking and molecular dynamics simulation

SDF files for the ligands' structures were obtained from PubChem.^25^ The PDB file for PIK3CA's protein structure was from RCSB Protein Data Bank (PDB).^26^ The AutoDock tools were used to process and prepare ligands and the receptor as directed by the manual. The AutoDock Vina was utilized to dock each ligand with the receptor, and the binding complexes with a docking score below −5 kcal/mol were considered to have successful binding. The binding sites of the binding complexes were analyzed using the PLIP platform.^27^ PyMol (DeLano Scientific) was used to visualize the results of AutoDock Vina and PLIP. To investigate the binding patterns of CN and CD to PIK3CA targets, MD simulations were performed, utilizing the docking structures of CN and CD to PIK3CA as the designed coordinates. The ff99SB^28^ and Generalized Amber Force Field (GAFF) force fields^29^, ^30^ were utilized for the receptor and ligand, respectively. Every system was dissolved in a truncated octahedral box of TIP3P water molecules with an edge distance of 10 Å. A 30‐ns production simulation run was carried out with the NPT combination at 310 K and 1 atm. Coordinate traces were recorded at 1‐ps intervals throughout the MD run. AMBER 1241^31^ was used to perform the MD simulations.

### 2.2 HEI‐OC1 cells and treatments

HEI‐OC1 cells were incubated in high‐glucose DMEM (Gibco, Cat.no. 11966025) with 5% fetal bovine serum (FBS, Gibco, Cat.no. 26140079) without antibiotic addition under appropriate conditions (33°C, 5% CO_2_). In all experiments, each group of cells was pretreated with CN or CD (Selleck; S2283 and S2282, respectively) for 2 h before treatment with cisplatin (Sigma‐Aldrich, Cat.no. 479306) at 30 μM for another 24 h.

### 2.3 Mouse cochlear explant culture

Neonatal C57BL/6J mice were decapitated after anesthesia. Cochleae were isolated in phosphate‐buffered saline (PBS), then laid on glass coverslips coated with Cell‐Tak (BD Biosciences, Cat.no. 354240) on ice. Then, cochlear explants were cultured overnight in DMEM/F12 growth medium containing N2/B27 (Invitrogen) and 50 IU/mL ampicillin at 33°C and 5% CO_2_.

### 2.4 Immunofluorescent staining

The treated cochlear explants underwent a 30‐min fixation with 4% paraformaldehyde (PFA, pH 7.4) in PBS. Cochlear explants were then permeabilized with 1% Triton X‐100 in PBS (PBST, 30 min) and blocked with 10% donkey serum in PBST (1 h). The tested primary antibodies were directed against parvalbumin (1:500, Abcam), myosin 7a (1:500, Proteus Biosciences), cleaved caspase‐3 (1:500, Cell Signaling Technology), and TUJ‐1 (1:1000, Biolegend). Incubation with the diluted primary antibodies was performed overnight at 4°C, followed by three PBS washes and then incubation with corresponding fluorescent secondary antibodies. 4′,6‐diamidino‐2‐phenylindole (DAPI; Sigma) counterstaining was carried out for 10 min at room temperature, and fluorescence was observed under a Leica SP8 laser scanning confocal microscope (Leica Microsystems).

### 2.5 TUNEL assay

To assess apoptosis in cultured cochlear explants, DNA fragmentation was evaluated by the TUNEL assay. After processing, the explants underwent incubation for 30 min at 37°C shielded from light according to the TUNEL staining instructions (Roche, Cat.no. 11684795910). Images of all cochlear explants were acquired under a Leica TCS SP8 confocal laser microscope (Leica Microsystems).

### 2.6 Cell viability assay

Cell viability assessment was conducted utilizing Cell Counting Kit‐8 (CCK‐8, Sigma, Cat.no. 96992). HEI‐OC1 cells seeded at 5000/well in 96‐well plates underwent culture at 33°C with 5% CO_2_. Drugs were added as described previously, and five replicates were established for each group. After drug treatment, the CCK‐8 solution was added for a 4‐h incubation, and optical density was read on a microplate reader (Bio‐Rad) at 450 nm.

### 2.7 ROS detection

Cochlear explants were assessed for reactive oxygen species (ROS) production with MitoSox Red (Invitrogen, Cat.no. M36008), a fluorescent superoxide indicator that selectively targets mitochondria. Cochlear explants were incubated as described above and stained with 5 μM MitoSox Red for 15 min at 37°C in a humidified environment with 5% CO_2_. The samples were then fixed, permeabilized, and blocked as described above, with subsequent incubation with an anti‐myosin 7a antibody. Finally, the stained cochlear explants were imaged under a Leica TCS SP8 microscope (Leica Microsystems). HEI‐OC1 cells treated as described above underwent a 30‐min incubation with 5 μM MitoSox Red. Following three PBS washes, HEI‐OC1 cells underwent imaging by fluorescence microscopy, and fluorescent intensity was assessed by flow cytometry assay.

### 2.8 Mitochondrial membrane potential detection

Mitochondrial membrane potential (ΔΨm) was assessed by staining with tetramethyl rhodamine methyl ester perchlorate (TMRM; Invitrogen, Cat.no. T668). After HEI‐OC1 cell treatment as described previously for different groups at 33°C, 5% CO_2_ for 24 h, staining was performed with 20 nM TMRM for 30 min. Image acquisition was performed under a Leica TCS SP8 microscope (Leica Microsystems), and quantitative analysis was carried out flow cytometric with analysis software.

### 2.9 Immunoblot

Treated HEI‐OC1 cells in various groups underwent lysis on ice with the RIPA buffer (Protein Biotechnology, PP109) supplemented with a protease inhibitor cocktail (Sigma, 04693132001). The lysates were cleared by centrifugation (12,000 × *g*, 15 min) at 4°C. The resulting supernatants were examined for protein quantitation with the BCA protein kit (Beyotime, P0010S). After protein separation by 12% SDS‐PAGE, the bands were electro‐transferred onto nitrocellulose membranes. Next, a 1‐h blocking was carried out with 5% skimmed milk in Tris‐buffered saline (TBST) with 0.1% Tween‐20. This was followed by successive incubations with primary (1:500; 4°C overnight) and HRP‐linked anti‐rabbit secondary (1:2000; Super Signal West) antibodies. The applied primary antibodies targeted GAPDH, AKT (1:500; Cell Signaling Technology, 4691 s), PI3K (1:500; CST, 4257 s), phospho‐AKT at Ser473 (1:500; CST, 9271), phospho‐PI3 Kinase p85 (Tyr458)/p55 (Tyr199) (1:500; CST, 4228), cleaved caspase‐3 (1:500; CST, 9664 s), and caspase‐3 (1:500; CST, 9662 s). Visualization utilized an enhanced chemiluminescence detection system. ImageJ (Broken Symmetry Software, National Institutes of Health) was employed for data analysis.

### 2.10 Cochlear HCs and SGNs counting

The numbers of HCs were recorded in the apical, middle, and basal turns for each cochlea separately. The amounts of HCs in various groups were recorded in units of 100 μm at different turns and averaged. Assays were repeated thrice. For quantitative assessment of SGN, immunolabeling of SGN was performed with anti‐TUJ‐1 antibody. SGN soma density per unit area (10,000 μm^2^) and neurite density per unit length (100 μm), as well as neurite growth length directly measured by the TUJ‐1‐labeled nerve fiber (unit: μm), were calculated and averaged for each group of 10 samples.

### 2.11 Statistical analysis

Data analysis utilized GraphPad Prism (version 6; GraphPad Software). The normality test was performed through D'Agostino & Pearson omnibus normality test and Shapiro–Wilk normality test. Data were presented as mean ± SEM. One‐way ANOVA was performed for comparisons. Multiple comparisons were performed among the different groups. Bonferroni correction was used to limit the risk of a false‐positive inference. All the *p* values were adjusted, and *p* < 0.05 was deemed to reflect the statistical significance.

## 3 RESULTS

### 3.1 Molecular docking and dynamics simulation between CN/CD and PIK3CA

**FIGURE 1:**
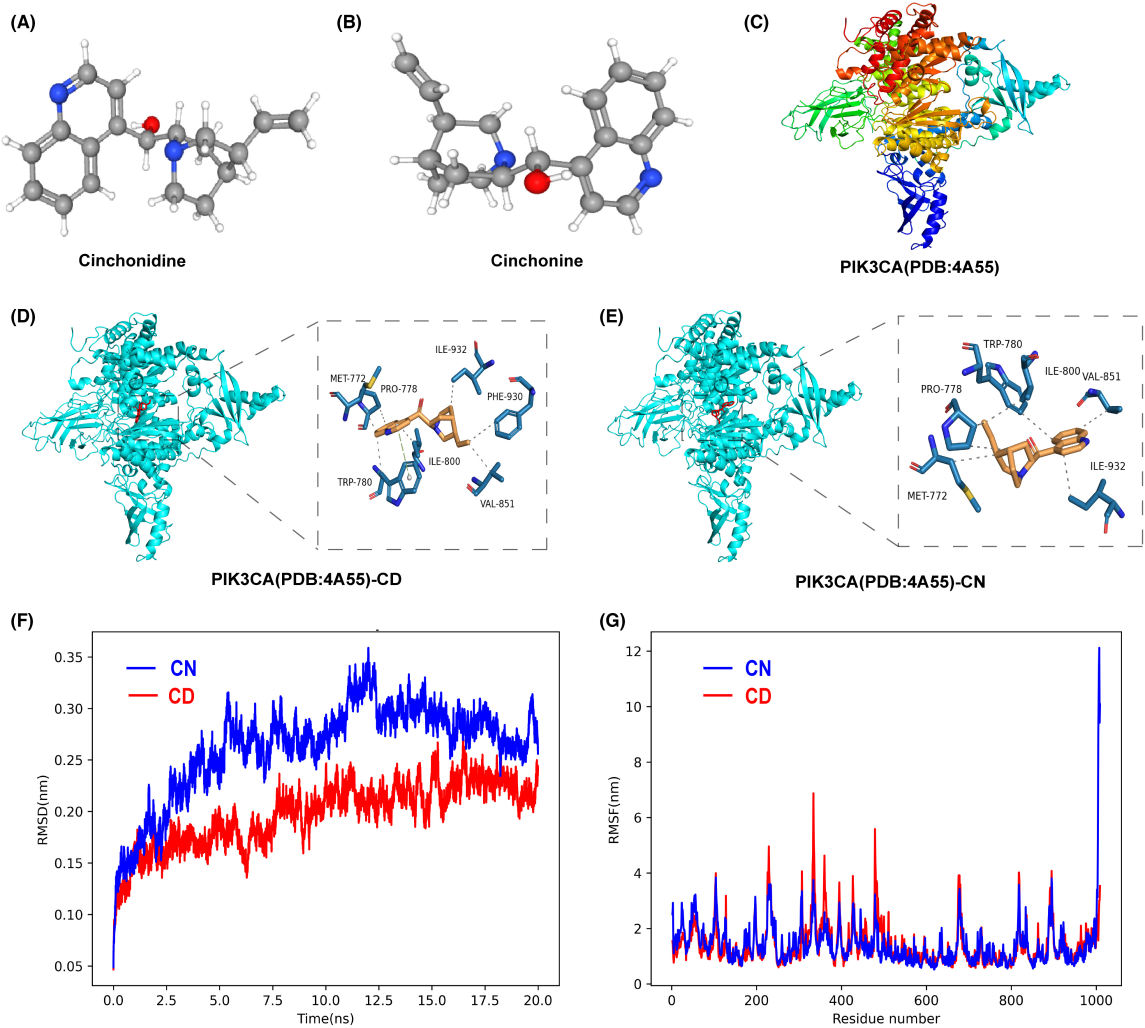
Molecular docking and dynamic analysis of CN or CD and PIK3CA. (A) 3D molecular structure of cinchonidine (CD). (B) 3D molecular structure of cinchonine (CN). (C) Structure of PIK3CA (PDB ID: 4a55). (D, E) Molecular docking between PIK3CA and CD or CN, and visualization of binding site residues. (F) RMSD of the CD‐PIK3CA and CN‐PIK3CA complexes for 20 ns. (G) RMSFs of CD‐PIK3CA and CN‐PIK3CA complex residues. The figures were generated using PyMOL.

We selected 17 drugs from a library of FDA‐approved natural herbs after semi‐flexible molecular docking targeted to PIK3CA (PDB ID: 4a55) using AutoDock Vina software and evaluated them by final interaction scores (Figure S1). Most of the selected small molecule compounds can dock to PIK3CA protein (Figure S2); among them, CN and CD scored the highest binding energy to PIK3CA protein (Table S1). The CN and CD binding patterns and sites for compound–target interactions are depicted in Figure 1A–E. We performed 20‐ns simulations of the complexes for estimating the stability of protein–ligand complexes under dynamic conditions. The root‐mean‐square deviation (RMSD) of the PIK3CA backbone was determined to assess the stability of the motion trajectories of the PIK3CA‐CN and PIK3CA‐CD complexes (Figure 1F). After 7.5 ns, the RMSD of PIK3CA‐CN and PIK3CA‐CD reached equilibrium and fluctuated around the mean value, with a mean value for the root‐mean‐square fluctuation below 0.1 nm. The root‐mean‐square fluctuation (RMSF) of protein atoms reflects the flexibility of the protein; the lower the RMSF value, the higher the stability (Figure 1G). These trajectories suggest that CN and CD could bind to PIK3CA and generate complexes with relatively stable conformations.

### 3.2 CN and CD prevent cisplatin‐associated HC loss in mouse cochlear explants

**FIGURE 2:**
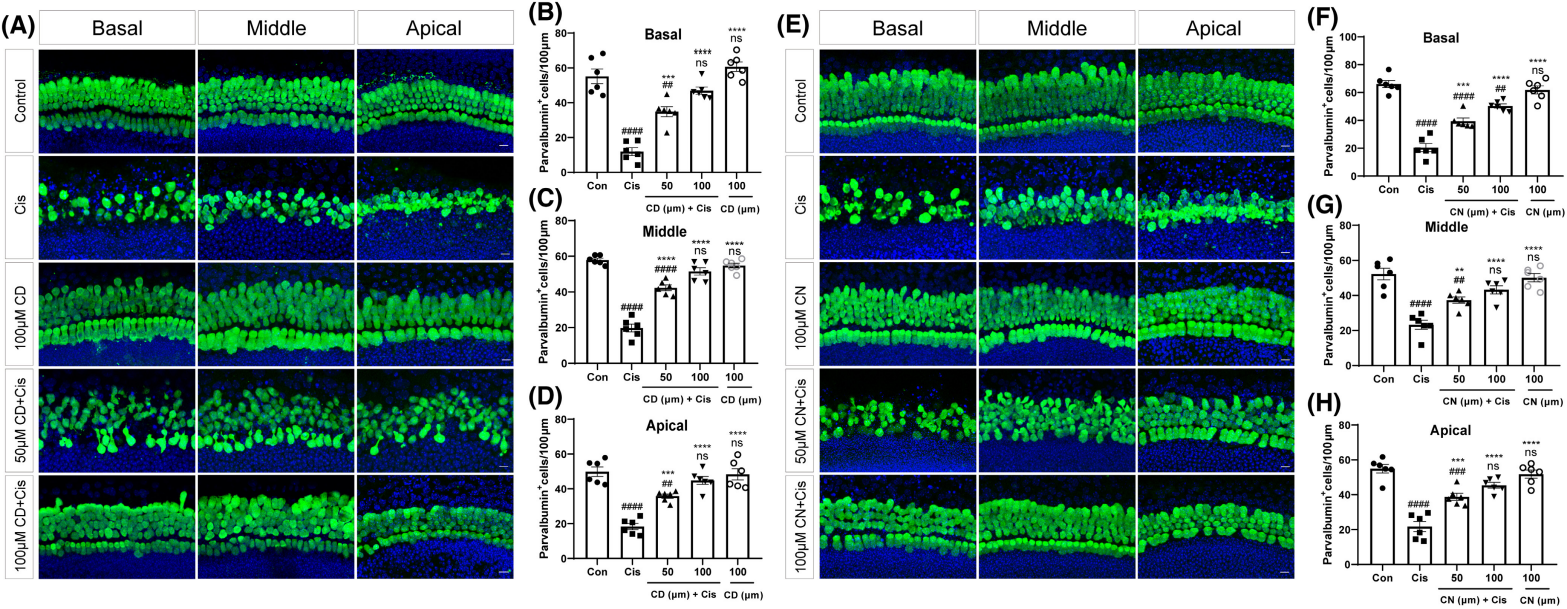
Effects of CN and CD on cisplatin‐injured cochlear HCs. (A and E) Images of cochlear explants labeled with parvalbumin (green) for various groups. Scale bar, 10 μm. (B–D, F–H) Quantitation of parvalbumin‐positive HCs at the base, middle, and apex of cochlear explants in various groups. The total amounts of HCs per 100 μm were determined. Data are presented as mean ± SEM. **p < 0.01, ***p < 0.001, ^****^ *p* < 0.0001 versus cisplatin group; ^##^ *p* < 0.01, ^###^ *p* < 0.001, and ^####^ *p* < 0.0001 versus control group; ns, non‐significant versus control group. *n* = 6/group.

To confirm whether CN and CD could exert protective effects on cochlear HCs and to determine the optimal concentration for drug action, we used neonatal mouse cochlear explant cultures in vitro. Immunofluorescence staining with parvalbumin antibody was used to characterize cochlear HCs (Figure 2A,E). HCs were markedly decreased after treatment with cisplatin alone, whereas 100 μM CN or CD pretreatment starkly elevated HC survival following cisplatin treatment (Figure 2B–D,F–H). Therefore, in the following experiments, we chose 100 μM CN or CD as the treatment condition for cochlear explants. Additionally, the number of parvalbumin‐positive HCs was comparable between CN or CD treatment alone groups and control groups without cisplatin damage (Figure 2A–H), indicating that CN or CD alone did not negatively affect HC survival. Interestingly, the level of HC loss in the basal turn was higher compared with those in the middle and apical turn (Figure 2B–D,F–H), corroborating previously reported findings that cisplatin‐induced hearing damage first affects the region of high‐frequency hearing (basal turn of cochlea).^32^

### 3.3 CN and CD protect HCs from cisplatin‐associated apoptosis in mouse cochlear explants

**FIGURE 3:**
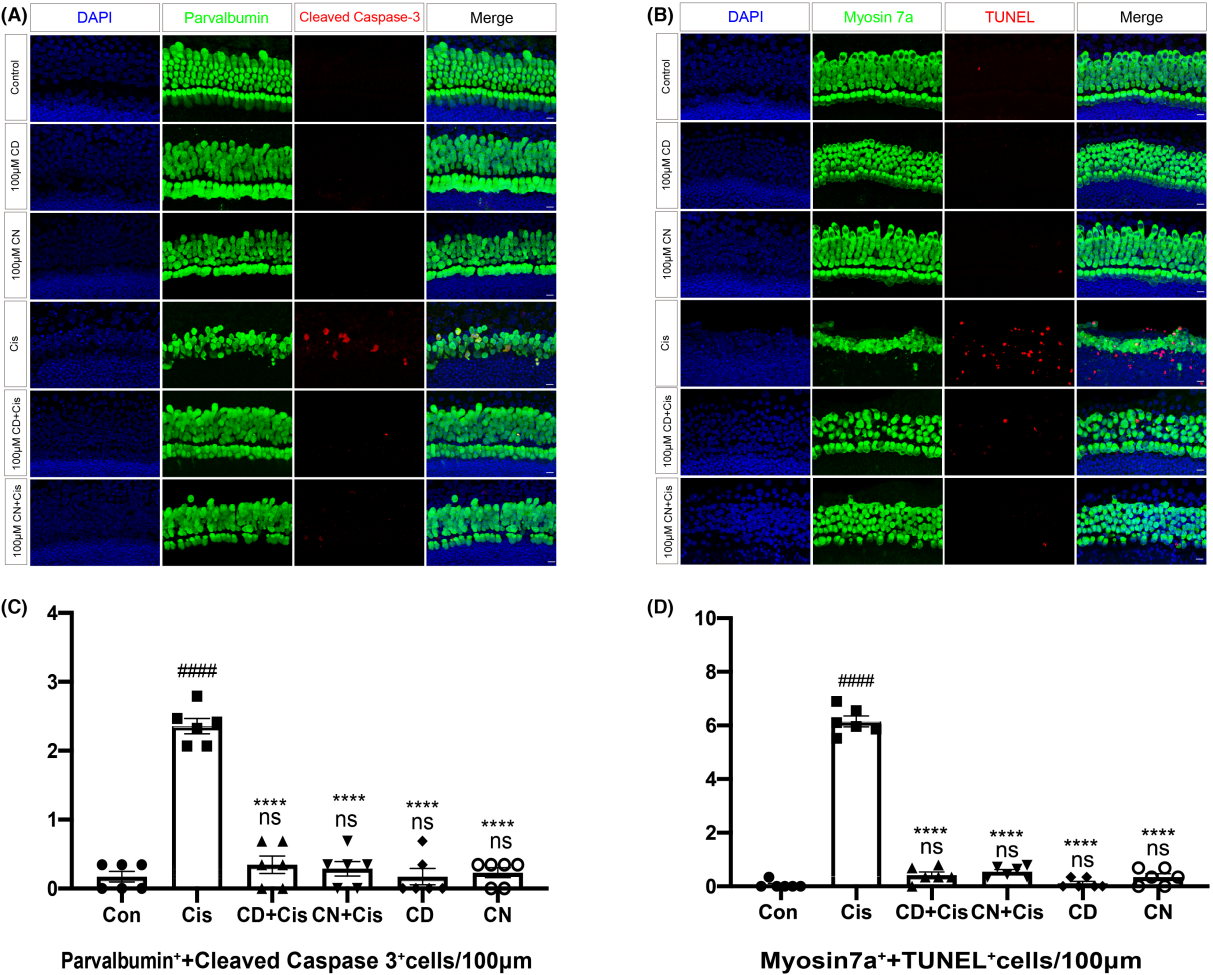
Effects of CN and CD on cochlear HC apoptosis. (A) Immunostaining of cleaved caspase‐3 (red) in the middle turn of cochlear explants from various treatment groups. HCs are labeled with parvalbumin (green). Scale bar, 10 μm. (B) Images of TUNEL staining (red) in the middle turn of cochlear explants from different groups. HCs are labeled with myosin 7a (green). Scale bar, 10 μm. (C) Quantitation of parvalbumin and cleaved caspase‐3 double‐positive HCs of the middle turn of cochlear explants for each group. (D) Quantitation of myosin 7a and TUNEL double‐positive HCs of the middle turn of cochlear explants for various groups. Data are recorded as mean ± SEM. ^####^ *p* < 0.0001 versus control group; ^****^ *p* < 0.0001 versus cisplatin group; ns, non‐significant versus control group, *n* = 6 per group.

After clarifying the protective features of CN and CD in cisplatin‐induced HC loss, we next aimed to determine whether CN and CD could reduce cisplatin‐mediated hair cell apoptosis. We assessed apoptotic HCs in the treated cochlear explants by immunohistochemical detection of cleaved caspase‐3 and TUNEL. The results demonstrated that cisplatin‐treated cochleae had more cleaved caspase‐3 (red fluorescence)‐positive HCs than the control cochleae free of cisplatin (Figure 3A). However, CN or CD pretreatment greatly decreased the number of cleaved caspase‐3‐positive HCs (Figure 3C). CN or CD pretreatment could also substantially reduce the increment of cisplatin‐induced TUNEL‐positive HCs (Figure 3B,D). These findings indicated that CN and CD could protect HCs from cisplatin‐induced apoptosis in the mouse cochlea.

### 3.4 CN and CD attenuate cisplatin‐induced oxidative stress in mouse cochlear explants

**FIGURE 4:**
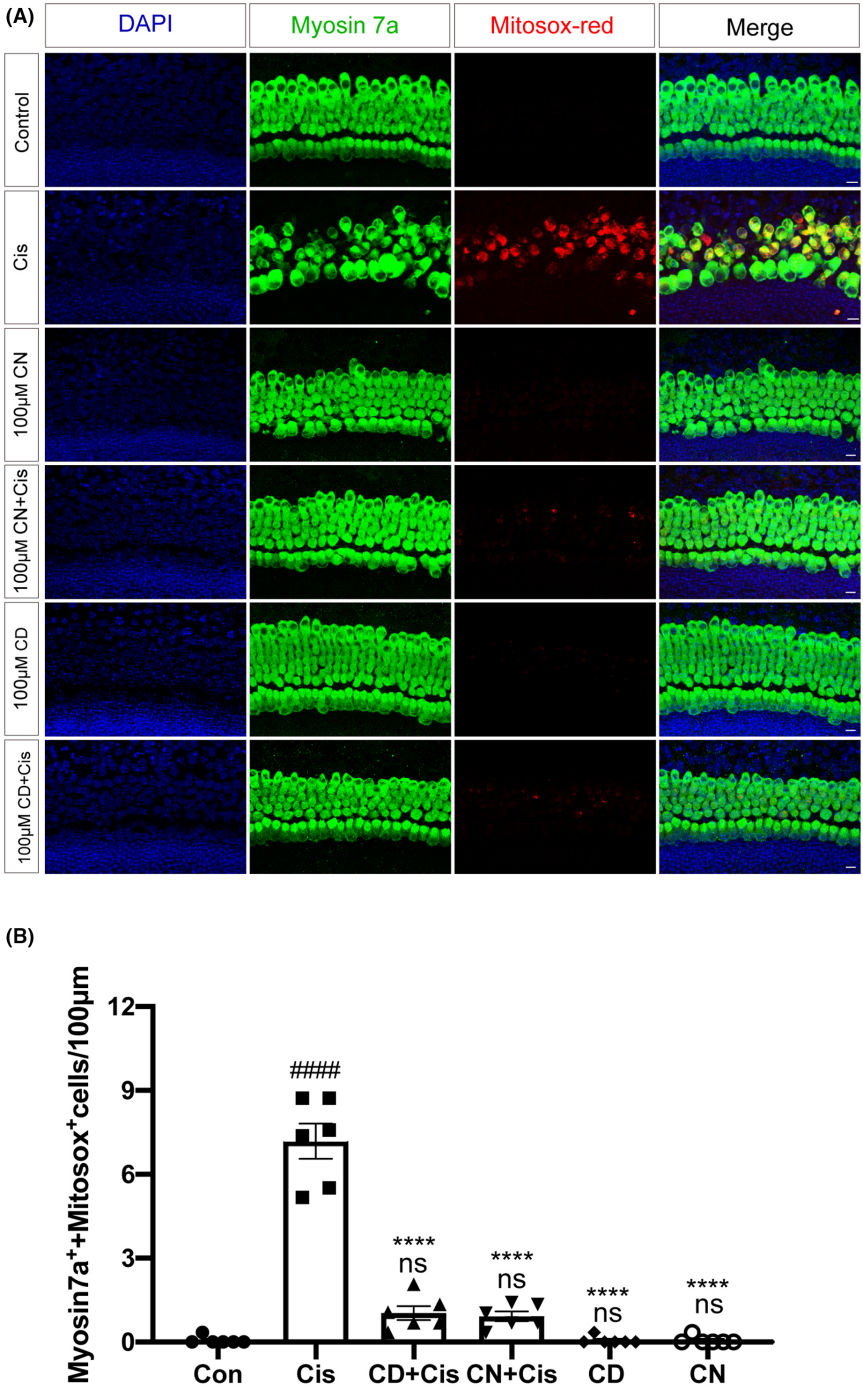
Effects of CN and CD on ROS levels in cochlear explants. (A) Staining of HCs from various groups of the middle turn, with MitoSox Red in red, myosin 7a in green, and DAPI in blue. Scale bar, 10 μm. (B) Quantitation of myosin 7a and MitoSox Red double‐positive HCs of the middle turn of cochlear explants for various groups. Data are shown as mean ± SEM. ^####^ *p* < 0.0001 versus control group; ^****^ *p* < 0.0001 versus cisplatin group; ns, non‐significant versus control group, *n* = 6 per group.

Excessive ROS production is the main factor causing cisplatin‐associated hair cell death and apoptosis,^33^ and mitochondria are one of the most important sites of ROS production. Therefore, ROS amounts were assessed in cisplatin‐induced cochlear explants by MitoSox Red immunofluorescence labeling. As shown in Figure 4A, the cisplatin‐alone exposure group had higher MitoSox Red‐positive HCs in comparison with the control samples without cisplatin, which had almost no red fluorescence, indicating that cisplatin treatment produced more ROS in cochlear explants. However, the number of MitoSox Red‐positive HCs in cochlear explants pretreated with CN or CD before cisplatin damage was reduced remarkably compared to the cisplatin‐alone group (Figure 4A,B), suggesting that CN or CD could prevent cisplatin‐related ototoxicity by diminishing excessive ROS accumulation. In addition, CN or CD alone had no effect on ROS amounts in comparison with the control group (Figure 4B).

### 3.5 CN and CD attenuated cisplatin‐related ototoxicity in HEI‐OC1 cells

**FIGURE 5:**
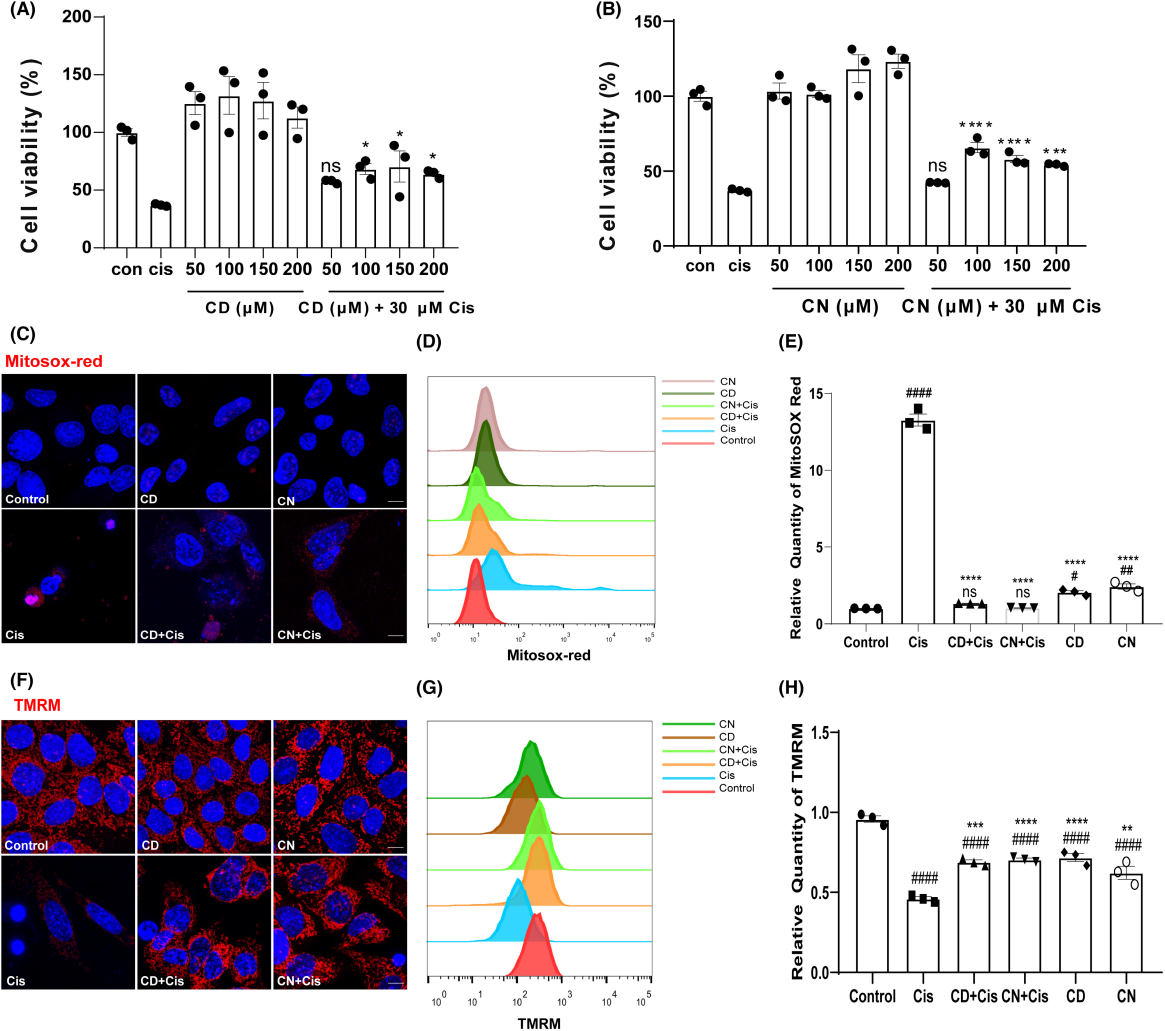
Effects of CN and CD on cisplatin‐triggered ototoxicity in HEI‐OC1 cells. (A, B) Cell viability in different treatment groups measured with CCK‐8 to determine the optimal drug concentration. Data are marked as mean ± SEM from three independent triplicate assays. *p < 0.05, ***p < 0.001, and ^****^ *p* < 0.0001 versus cisplatin only group. ns, non‐significant versus cisplatin only group. (C) Mitochondrial ROS levels are assessed by live cell staining with the MitoSox Red probe. (D, E) Quantitation of (C). Data are marked as mean ± SEM from three independent triplicate assays. ^#^ *p* < 0.1, ^##^ *p* < 0.01, ^####^ *p* < 0.0001 versus non‐treated control group; ^****^ *p* < 0.0001 versus cisplatin only group. ns, non‐significant versus non‐treated control group. Scale bar, 20 μm. (F) ΔΨm is assessed by staining live cells with the TMRM probe. (G‐H) Quantitation of (F). Data are marked as mean ± SEM from three independent triplicate assays. ^####^ *p* < 0.0001 versus non‐treated control group; **p < 0.01, ***p < 0.001, and ^****^ *p* < 0.0001 versus cisplatin only group. Scale bar, 20 μm.

To examine whether CN and CD protect HEI‐OC1 cells from cisplatin‐associated ototoxicity, cells were pretreated with CN or CD and then incubated with cisplatin. In the CCK‐8 assay, survival rates were indeed higher in CN and CD (50, 100, 150, and 200 μM) pretreated cells in comparison with the cisplatin‐alone group (Figure 5A,B). Based on these protective findings in different drug concentrations, we determined 100 μM for both CN and CD as the optimal protective concentration for further assays. We next investigated if CN and CD inhibited cisplatin‐related ROS overproduction in HEI‐OC1 cells. After collecting different groups of cells, MitoSox Red staining and flow cytometry were performed. As expected, cisplatin administration resulted in significantly higher MitoSox Red fluorescence intensity compared with untreated controls in HEI‐OC1 cells. By contrast, pretreatment with CN or CD before cisplatin markedly reduced the excessive ROS production triggered by cisplatin exposure (Figure 5C–E). The excessive ROS can reduce ΔΨm, as an earliest sign of impaired mitochondrial function, which eventually leads to apoptosis. We next assessed ΔΨm by TMRM staining and flow cytometry. As shown in Figure 5F, a 24‐h exposure of HEI‐OC1 cells to cisplatin decreased mitochondrial membrane potential in comparison with the untreated controls. This was reflected by a reduced proportion of red fluorescence (Figure 5G,H). The ΔΨm of cisplatin‐injured HEI‐OC1 cells was significantly increased after pretreatment with CN or CD. These results suggested that CN and CD increased cell survival, reduced ROS accumulation, and alleviated mitochondrial dysfunction in cisplatin‐damaged HEI‐OC1 cells.

### 3.6 CN and CD alleviate cisplatin‐induced SGN injury

**FIGURE 6:**
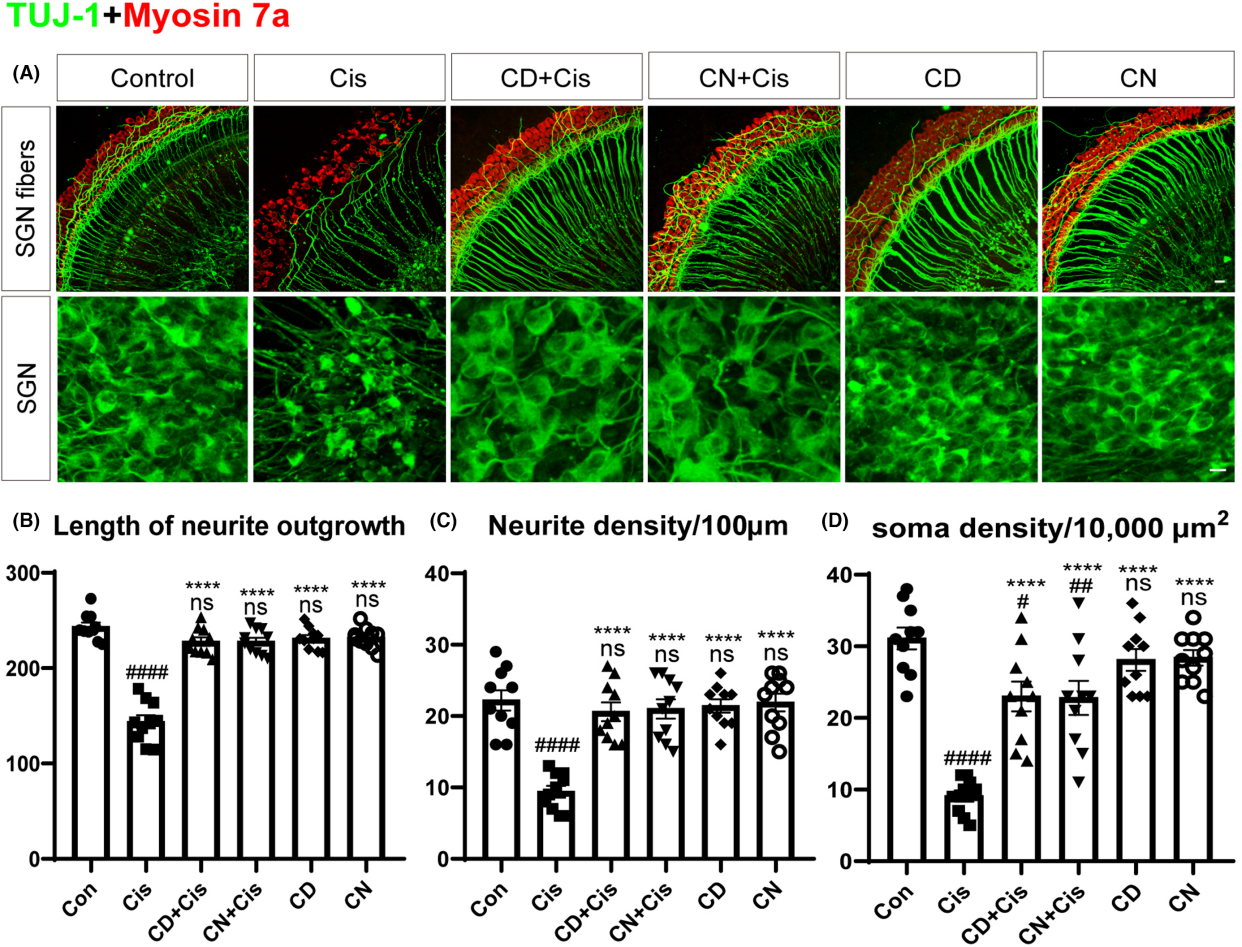
Effects of CN and CD on cisplatin‐triggered cochlear SGN damage. (A) Confocal micrographs of SGNs in various groups. SGNs are labeled with TUJ‐1 (green), and HCs are labeled with Myosin 7a (red). Scale bar, 20 μm. (B–D) Quantitation of SGN soma densities, neurite densities, and lengths of neurite outgrowth of the middle turns of cochlear explants in various groups. Data are shown as mean ± SEM. ^#^ *p* < 0.1, ^##^ *p* < 0.01, and ^####^ *p* < 0.0001 versus control group; ^****^ *p* < 0.0001 versus cisplatin group. ns, non‐significant versus control group. *n* = 10 per group.

To examine the protective effects of CN and CD on SGNs, we labeled peripheral auditory nerve fibers and SGN somata with TUJ‐1, stained cochlear HCs with myosin 7a, and assessed SGN injury and HC loss in various groups (Figure 6A). We found that the somatic cells of SGNs in the control and CN‐ or CD‐alone groups were large and round or oval, with strong TUJ‐1 signals and neatly arranged HCs. The nerve fibers were arranged in smooth and dense bundles extending radially from SGNs to HCs. There were markedly less SGNs and HCs in the cisplatin‐treated group; the remaining somata were shriveled, and nerve fibers were broken and arranged in an unorganized pattern. However, in the CN or CD pretreatment plus cisplatin groups, there were reduced loss of SGNs and HCs and larger somata in comparison with the cisplatin‐alone group (Figure 6A). Subsequently, the survival of SGNs was quantified. As depicted in Figure 6B–D, neurite densities, lengths of neurite outgrowth, and SGN soma densities were significantly reduced in the cisplatin group, while the degree of SGN damage was starkly lower in the CN or CD pretreatment group in comparison with the cisplatin‐alone group.

### 3.7 CN and CD reduce cisplatin‐associated injury by regulating PI3K‐AKT signaling

**FIGURE 7:**
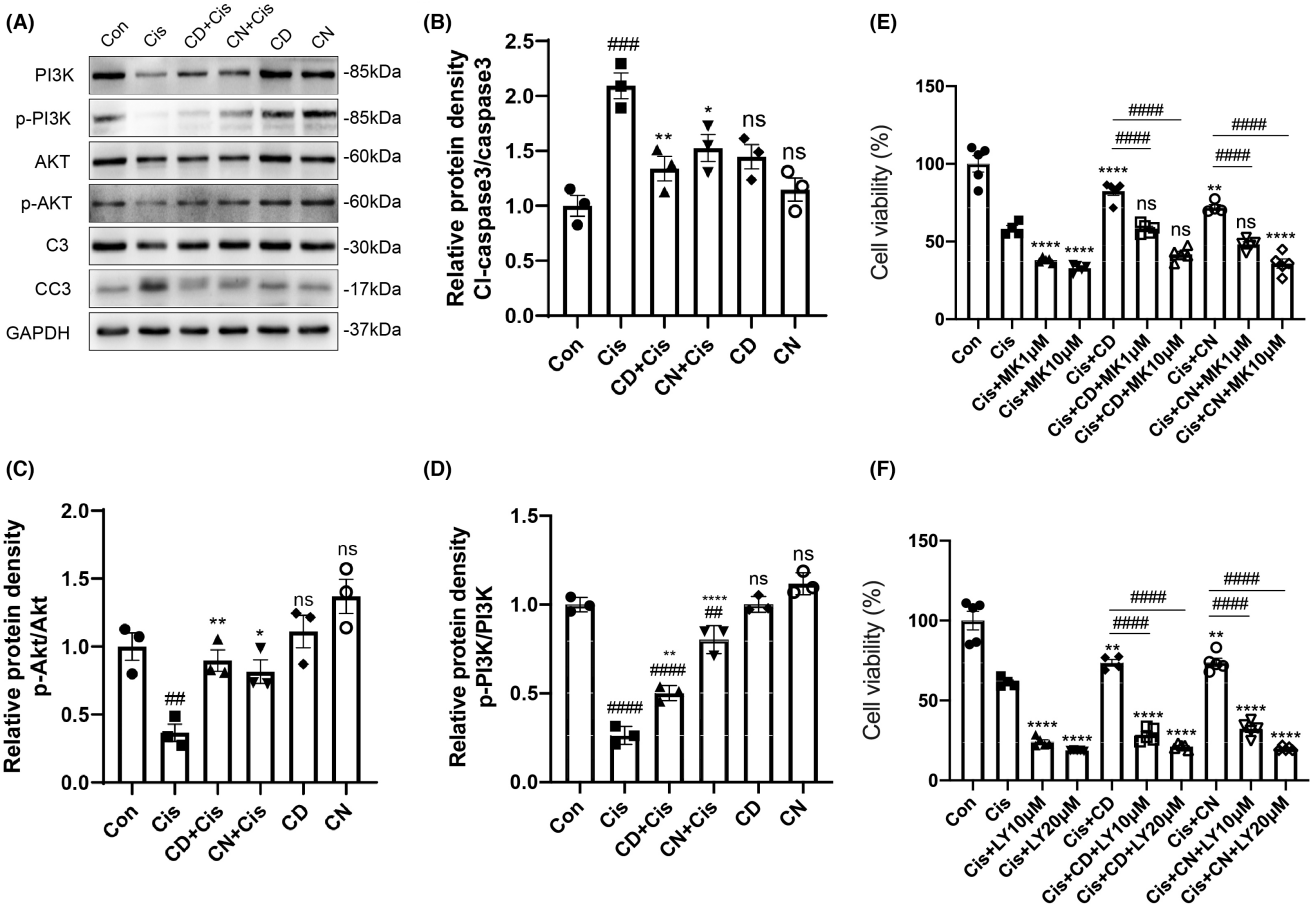
Effects of CN and CD on PI3K/AKT signaling. (A) PI3K, p‐PI3K, AKT, p‐AKT, caspase‐3 (C3), and cleaved caspase‐3 (CC3) protein amounts are tested by immunoblot. (B–D) Semi‐quantitative densitometric analysis of the respective blots of the proteins in (A). Data are mean ± SEM from three independent assays. ^##^ *p* < 0.01, ^###^ *p* < 0.001, and ^####^ *p* < 0.0001 versus control group; ns, non‐significant versus control group; *p < 0.05, **p < 0.01, and ^****^ *p* < 0.0001 versus cisplatin group. (E) HEI‐OC1 cells are pretreated with CD or CN and the AKT inhibitor MK‐2206 (1 or 10 μM) for 2 h and then exposed to 30 μM cisplatin for another 24 h. Cell survival is assessed with CCK‐8 assay. Data are mean ± SEM. **p < 0.1 and ^****^ *p* < 0.0001 versus cisplatin (30 μM) only group; ns, non‐significant versus cisplatin (30 μM) only group; ^####^ *p* < 0.0001 versus CD or CN and cisplatin treatment group. (F) Effect of the PI3K inhibitor LY294002 (10 or 20 μM) on CD or CN‐induced protection in HEI‐OC1 cells. Data are mean ± SEM. **p < 0.01 and ^****^ *p* < 0.0001 versus cisplatin (30 μM) only group; ^####^ *p* < 0.0001 versus CD or CN and cisplatin treatment group. MK, MK‐2206; LY, LY294002.

To unveil the molecular mechanisms by which CN and CD alleviate cisplatin‐associated ototoxicity, changes in PI3K‐AKT signaling effectors and apoptotic proteins were examined in different groups of HEI‐OC1 cells by immunoblot. We observed p‐PI3K and p‐AKT amounts were reduced upon treatment with cisplatin alone compared with untreated control cells (Figures 7A and S3). In contrast, these two proteins were upregulated in the CN + cisplatin and CD + cisplatin groups compared with cells administered cisplatin alone (Figure 7C,D). Additionally, cleaved caspase‐3 was starkly upregulated upon cisplatin treatment (Figure 7B). After pretreatment with CN or CD, these changes were significantly reversed. To further examine PI3K‐AKT pathway's role in the protective effects of CN and CD, HEI‐OC1 cells underwent pretreatment with specific PI3K (LY294002) and AKT (MK‐2206) inhibitors, respectively, for 2 h before cisplatin administration, and changes in cell viability were analyzed with CCK‐8 assay. The results indicated that PI3K and AKT suppression with LY294002 and MK‐2206, respectively, alleviated cell viability induction by CN or CD after cisplatin injury (Figure 7E,F). These results suggest that CN and CD provided an anti‐apoptotic effect on cisplatin‐induced injury in HEI‐OC1 cells by regulating the PI3K‐AKT pathway.

## 4 DISCUSSION

The current work showed that cisplatin activated the mitochondrial apoptotic pathway and caused ROS overproduction, leading to HC apoptosis, which was corroborated to previously reported findings.^34^ We then demonstrated that pretreatment with CN or CD increased cell viability in HEI‐OC1 cells upon exposure to cisplatin. CN and CD could reduce apoptotic pathway activation in the mitochondria and ROS accumulation. Furthermore, utilizing mouse cochlear explants, the protective features of CN and CD in cisplatin‐related ototoxicity were verified in vitro. In addition, PI3K‐AKT signaling was shown to contribute to the protective effects of CN and CD on HEI‐OC1 cells.

HC apoptosis is considered the major mechanism of cisplatin‐related ototoxicity, which is associated with ROS‐related mitochondrial damage.^35^ Currently, many antioxidant and anti‐apoptotic drugs are known to regulate the ROS pathway and protect HC from damage.^36^, ^37^ Here, we examined ROS production with MitoSox Red, a mitochondria‐specific ROS indicator, mitochondrial membrane potential with TMRM, and apoptosis with the apoptosis indicators cleaved caspase‐3 and TUNEL. We found administration of CN or CD significantly reduced mitochondrial ROS and prevented mitochondrial dysfunction and apoptosis in HC after cisplatin injury.

Increasing evidence suggests that ROS overproduction is associated with many intracellular pathways, including PI3K‐AKT signaling. Recent reports indicate that PI3K‐AKT signaling is important in HC survival. Kanamycin suppresses the PI3K‐AKT survival pathway, causing OHC death.^38^ When cochlear explants co‐administered with gentamicin and PI3K inhibitors, HC damage markedly increases in comparison with treatment with gentamicin alone.^39^ PI3K‐AKT signaling might play the most critical role in the mechanism by which RG108 protects HEI‐OC1 cells from apoptosis caused by cisplatin.^40^ Here, we performed molecular docking and dynamics assessment of CN and CD targeting to PIK3CA; the results showed that CN and CD could bind to PIK3CA and produce stable complexes. Furthermore, experiments showed that in HEI‐OC1 cells, cisplatin administration resulted in decreased levels of phosphorylated AKT and PI3K, accompanied by increased apoptosis‐associated proteins. Interestingly, LY294002 and MK‐2206 (PI3K and AKT inhibitors, respectively) increased cisplatin‐related ototoxicity in HEI‐OC1 cells and partially blocked the protective effects of CN and CD, respectively, further confirming the involvement of PI3K‐AKT signaling in CN and CD mediated HEI‐OC1 cell protection. The above findings confirmed that CN and CD reduced cisplatin ototoxicity in HEI‐OC1 cells by regulating PI3K‐AKT signaling. However, we cannot exclude that CN and CD may protect HCs from cisplatin ototoxicity by regulating other signaling pathways, which offers future study directions.

Since some mechanisms of cisplatin‐related ototoxicity also contribute to anticancer activity, protective drugs such as antioxidants may also impact cisplatin‐mediated antitumor effects.^10^, ^41^ Furthermore, studies should be conducted to determine whether CN and CD affect the anticancer effect of cisplatin while preventing hearing loss clinically. These conclusions were based on in vitro models including HEI‐OC1 cells and cochlear explants and should be validated in the future using an in vivo mouse model of deafness, for instance.

In summary, we confirmed that CN and CD pretreatment could prevent cisplatin‐related ototoxicity in HEI‐OC1 cells and mouse cochlear explants. CN and CD inhibited cisplatin‐triggered activation of apoptotic pathways and ROS overproduction. Furthermore, we demonstrated an important role of the PI3K‐AKT pathway in CN and CD resistance to cisplatin‐induced ototoxicity. In summary, our current study suggests that CN and CD could be potential agents for protection against the ototoxicity of cisplatin.

## AUTHOR CONTRIBUTIONS

**Zhiyuan Zhang** and **Yingzi He**: Conceptualization, methodology, writing—original draft. **Dongmei Tang**, **Zhiyuan Zhang**, and **Yingzi He**: Funding acquisition. **Dongmei Tang**, **Xue Wang**, **Yimeng Li**, and **Jingfang Wu**: Writing—review and editing, project administration, methodology, formal analysis, validation. **Cai Li**, **Xiangyun Qiao**, **Li Fan**, **Yutao Chen**, and **Huanhuan Zhu**: investigation and formal analysis. All authors read and approved the final manuscript.

## FUNDING INFORMATION

This work was supported by grants from the National Natural Science Foundation of China (Nos. 82271158 and 82071045), Province Key Research and Development Program Project (No. GJJ190028), and Shanghai Rising‐Star Program (No. 23QC1401200).

## CONFLICT OF INTEREST STATEMENT

The authors declare that they have no competing interests.

## Supporting information


Figure S1.

Table S1.

## Data Availability

All data generated or analyzed during this study are included in this published article and its supplementary information files.
