# Correction to “Cinchonine and Cinchonidine Alleviate Cisplatin‐Induced Ototoxicity by Regulating PI3K‐AKT Signaling”

**DOI:** 10.1111/cns.70228

**Published:** 2025-01-20

D. Tang, X. Wang, J. Wu, Y. Li, C. Li, X. Qiao, L. Fan, Y. Chen, H. Zhu, Z. Zhang, and Y. He, “Cinchonine and Cinchonidine Alleviate Cisplatin‐Induced Ototoxicity by Regulating PI3K‐AKT Signaling,” *CNS Neuroscience & Therapeutics* 30, no. 2 (2024): e14403, https://doi.org/10.1111/cns.14403.

Figure 2A (100 μM CD, Middle; parvalbumin‐positive hair cells in the middle turn of cochlear explants) and Figure 3A (100 μM CD, Parvalbumin; parvalbumin‐positive hair cells in the middle turn of cochlear explants) were repeated accidentally. As detailed above, the image replication came from the same experimental conditions (the same group and the same fluorescent label), so this inadvertent replication did not change our experimental design or affect our conclusions. Also, we have provided a new version of Figure 2 with the necessary corrections below.

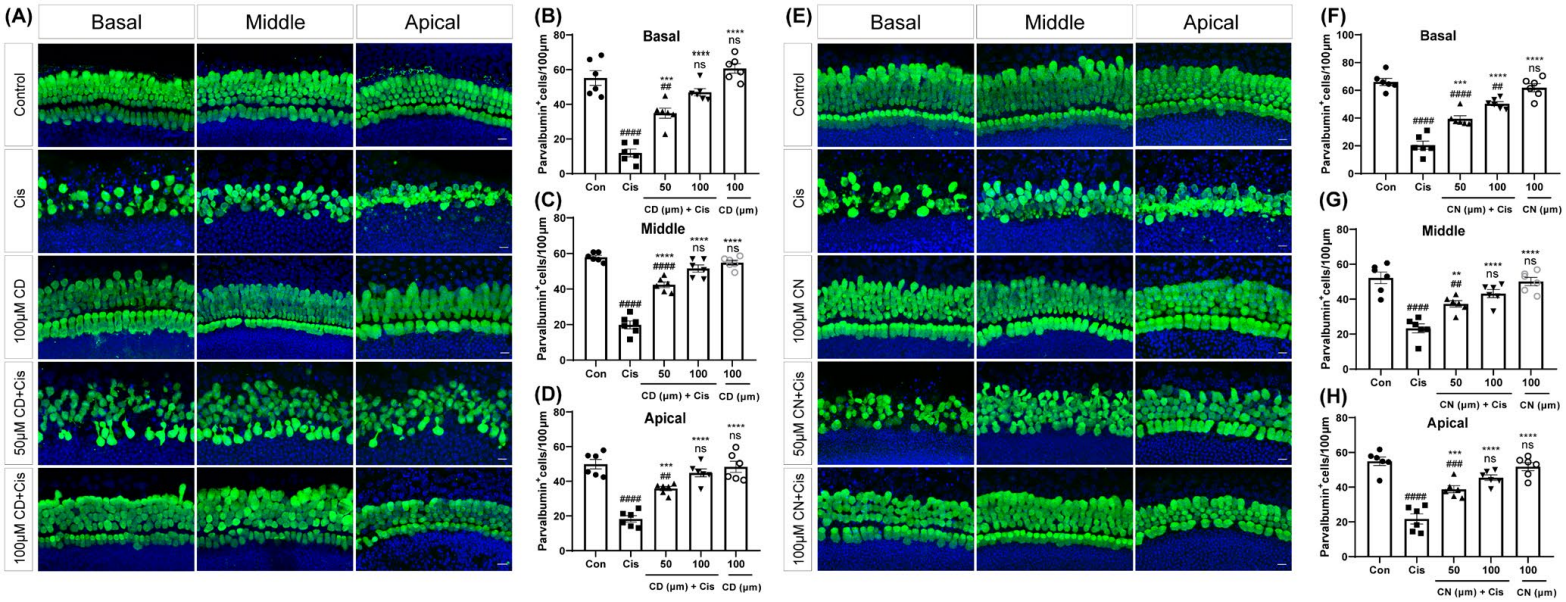

We apologize for this error.

