# Supplementary material for: Cinchonine and cinchonidine alleviate cisplatin‐induced ototoxicity by regulating PI3K‐AKT signaling
Source: CNS Neurosci Ther. 2023 Aug 14;30(2):e14403. doi: 10.1111/cns.14403 (PMC10848099; doi:10.1111/cns.14403)
Supplement: Supplementary file 1 — Figure S1. [file CNS-30-e14403-s002.docx]

**Supplementary Material**


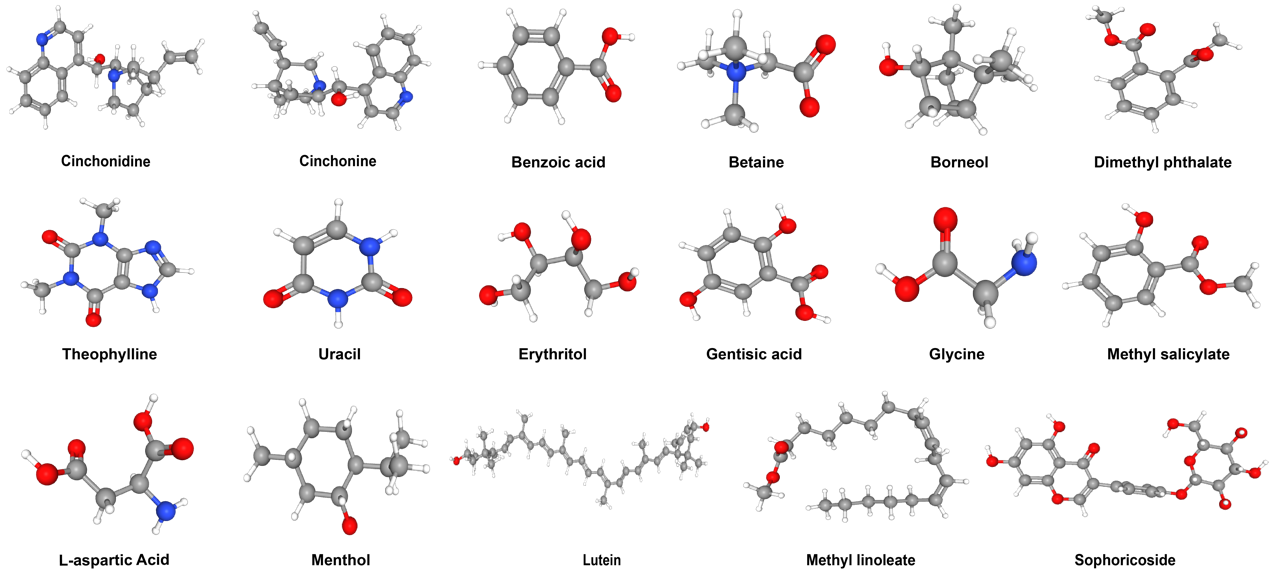


**Figure S1.** 3D structures of molecular compounds from FDA-approved Chinese drugs.


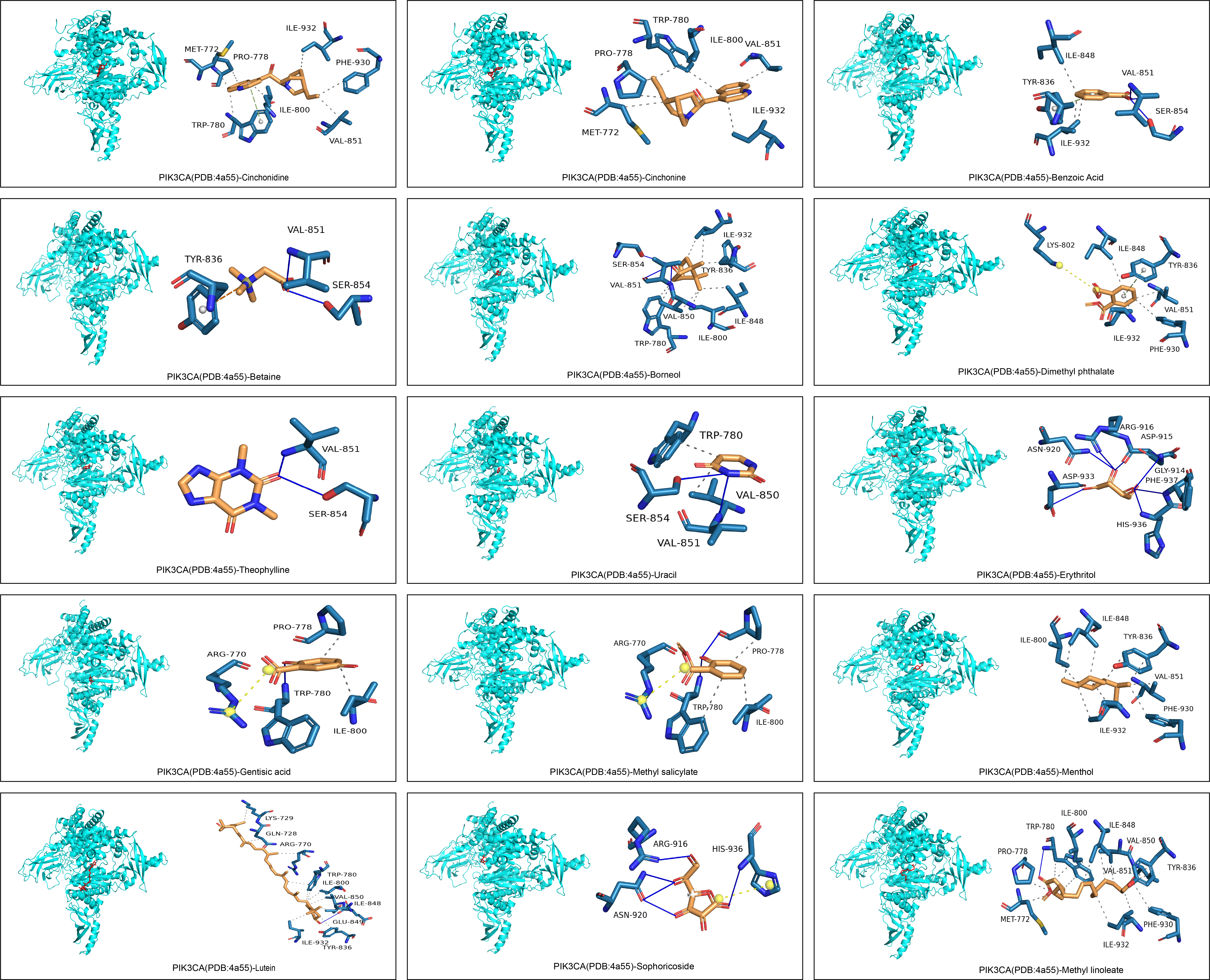


**Figure S2****.** Cartoon representation of intermolecular interactions between PIK3CA and molecular compounds and visualization of the binding site residues.


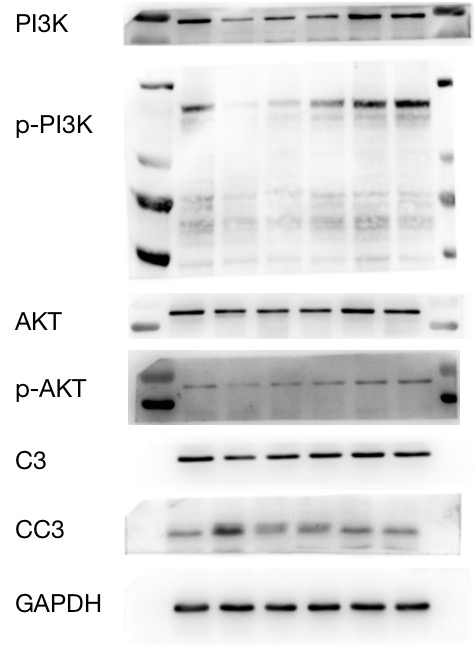


**Figure S3.** The original gel/blot images of Figure 7.
