# Supplementary material for: Cinchonine and cinchonidine alleviate cisplatin‐induced ototoxicity by regulating PI3K‐AKT signaling
Source: CNS Neurosci Ther. 2023 Aug 14;30(2):e14403. doi: 10.1111/cns.14403 (PMC10848099; doi:10.1111/cns.14403)

**Cinchonine and Cinchonidine alleviate cisplatin-induced ototoxicity by regulating PI3K-AKT signaling**


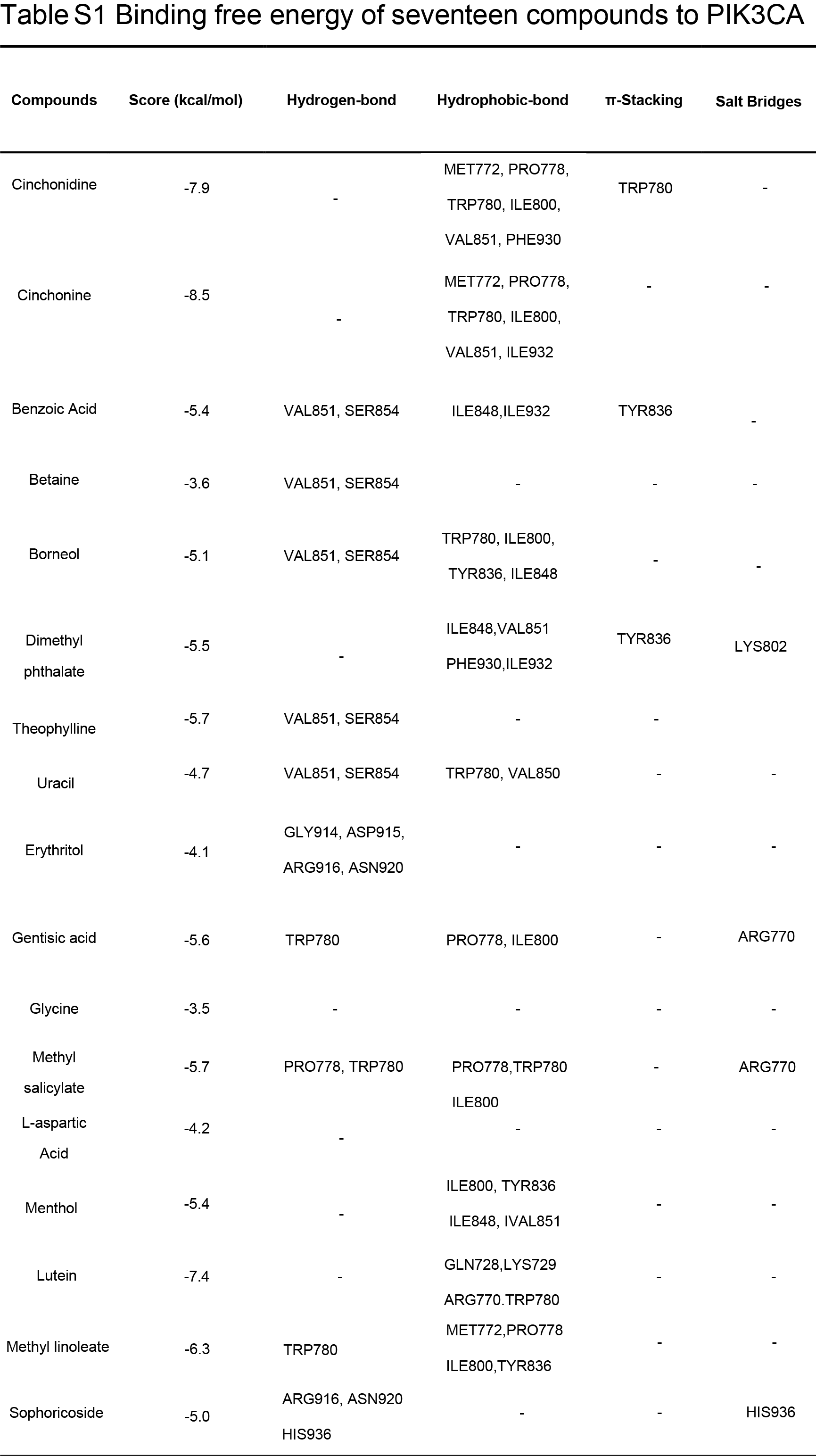

Supplement: Supplementary file 2 — Table S1. [file CNS-30-e14403-s001.docx]
